# Severe hyperkalemia following colon diversion surgery in a patient undergoing chronic hemodialysis: a case report

**DOI:** 10.1186/1752-1947-7-207

**Published:** 2013-08-14

**Authors:** Nina Kononowa, Michael J Dickenmann, Min Jeong Kim

**Affiliations:** 1 Clinic for Transplantation Immunology and Nephrology, University Hospital Basel, Basel, Switzerland

## Abstract

**Introduction:**

Potassium (K^+^) homeostasis in healthy subjects is maintained mainly by urinary excretion of K^+^. In patients with end-stage renal disease, the intestinal tract might assume an accessory K^+^ excretory role in the face of declining renal excretory function. Here, we report the case of a patient with end-stage renal disease who developed severe hyperkalemia following colon diversion surgery.

**Case presentation:**

A 56-year-old Caucasian woman undergoing hemodialysis experienced ischemic colitis, leading to ileocecal resection and a temporary ileostomy. She made a good recovery and her dietary intake improved. However, her pre-dialysis serum K^+^ level three weeks later was 7.2mmol/L, which was much higher than her previous level (range 4.9 to 6.1mmol/L). Despite dietary restriction of K^+^ and use of oral cation-exchange resin and low K^+^ dialysate, her serum K^+^ level remained high (6.1 to 8.3mmol/L). Six months later, her bowel continuity was restored and her serum K^+^ decreased to the previous level. Her fecal K^+^ concentration before and after stoma reversal showed a marked difference: 23mmol/L before and 60mmol/L after.

**Conclusions:**

We assume that the severe hyperkalemia seen in our patient was caused by reduced colonic K^+^ secretion due to the colon diversion. Our patient’s case demonstrates the importance of colonic K^+^ secretion for the maintenance of K^+^ homeostasis in patients with end-stage renal disease.

## Introduction

Potassium (K^+^) is an essential dietary mineral and major intra-cellular cation. It constitutes the main intra-cellular electrolyte and osmolyte necessary for fundamental processes such as membrane excitability, ion and solute transport or cell volume regulation [1]. Homeostatic maintenance of plasma K^+^ is therefore a critical physiological function. Total body exchangeable K^+^, measured with the use of radioactive K, averages 46mEq/kg in men and 39mEq/kg in women. Only 1.5 to 2.5 percent of total body K^+^ (about 65mEq) is found in the extracellular fluid [2]. Under conditions of a normal dietary K^+^ intake (80 to 100mmol per day), about 90 percent of dietary K^+^ is absorbed in the small intestine and an equivalent amount of the absorbed K^+^ is excreted mainly by the distal tubules of the kidney (about 90mmol per day). The contribution of the colon to net K^+^ absorption and secretion is trivial, and fecal K^+^ averages about 10mmol per day in healthy subjects [3].

During the development of end-stage renal disease (ESRD), many patients remain normokalemic for long periods, although renal excretory function deteriorates progressively. This can be explained by an increase in the K^+^ secretory capacity of remaining functional renal tubules, an adaptive response dependent on enhanced K^+^ uptake across the basolateral membrane, which is mediated by increased cortical and outer medullary Na^+^/K^+^-adenylpyrophosphatase (ATPase) activity [4]. However, this response cannot entirely explain the maintenance of K^+^ homeostasis in such patients, because urinary K^+^ excretion is generally substantially lower than in healthy individuals [5]. Metabolic studies indicated increased fecal K^+^ losses in patients with ESRD, raising the possibility that the intestinal tract might assume an accessory K^+^ excretory role in the face of declining renal excretory function [6,7].

Here, we report on an unusual case of severe hyperkalemia following ileocecal resection and a temporary ileostomy in a patient undergoing chronic hemodialysis (HD), and discuss the possible pathophysiological mechanisms to account for the clinical picture.

## Case presentation

A 56-year-old Caucasian woman with ESRD on regular HD was admitted to the hospital for an elective transplant nephrectomy. She initially developed ESRD at the age of 28 years after having idiopathic membranous nephropathy. In the next 28 years, she underwent two HD periods and two deceased-donor kidney transplantations, until she returned to chronic HD approximately one year before her current admission. As a preparation for a new transplantation, a transplant nephrectomy was planned. Her other relevant medical history included hypertension and coronary three-vessel disease.

**Figure 1:**
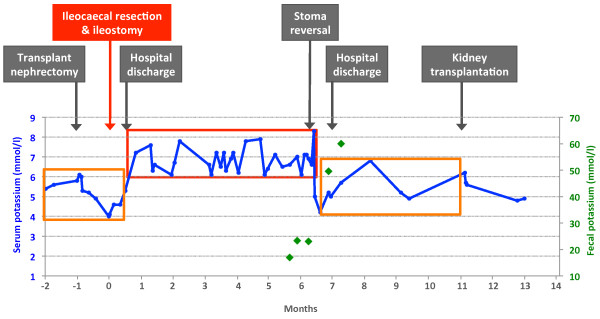
**Changes in serum potassium concentration before and after stoma operation.** The diamond symbols show the fecal potassium concentration before and after stoma reversal.

The post-operative course after the bilateral transplant nephrectomy was complicated, with an acute coronary syndrome on post-operative day six and an ischemic colitis with hematochezia on day 11. The colonoscopy performed on day 12 showed colitis lesions in the cecum, consistent with ischemic colitis. Over the following two weeks, she experienced repeated hematochezia despite repeated intra-mucosal adrenaline injections and endoclipping of bleeding vessels. Therefore, a resection of the ileocecum with a temporary ileostomy was performed on post-operative day 30. She made a good recovery and her dietary intake slowly improved over the following weeks. However, her routinely measured pre-dialysis serum K^+^ three weeks later was 7.2mmol/L. She had no remarkable symptoms or signs of hyperkalemia and the results of an electrocardiogram showed no hyperkalemia-related changes. Her previous pre-dialysis K^+^ level ranged between 4.9 and 6.1mmol/L (Figure 1). The results of blood gas analysis showed no evidence of severe metabolic acidosis as a potential cause of hyperkalemia (Table 1). Although her dietary intake of K^+^ was restricted, an oral cation exchange resin (sodium polystyrol sulfonate) was administered, and a low potassium dialysate was applied for the dialysis, her pre-dialysis serum K^+^ values in the next months remained high (6.1 to 8.3mmol/L).

**Table 1:** Changes of metabolic status before and after stoma operation

**Blood gas analysis**	**Without stoma**		**With stoma**		**After stoma reversal**	
pH	7.30	7.39	7.38	7.31	7.37	7.31
Active bicarbonate, mmol/L	19.4	19.6	21	18.6	19.9	20.1
Potassium, mmol/L	5.4	4.6	6.3	6.9	5.7	5.5

Six months later, her bowel continuity was successfully restored. Interestingly, pre-dialysis serum K^+^ values returned to their previous level without changes in diet, medication or dialysis regimen. In the following four months until the third deceased donor kidney transplantation, her serum K^+^ values remained similar to the previous range before ileostomy. Since we hypothesized that the colon diversion through ileostomy and therefore reduced colonic K^+^ secretion contributed to the development of severe hyperkalemia, we measured fecal K^+^ concentration before and after the restoration of bowel continuity (Table 2). The fecal K^+^ concentration before stoma reversal was 23.4 and 23.1mmol/L on two separate occasions. Under the treatment with an oral cation exchange resin, K^+^ concentration was slightly lower (17.0mmol/L). After the stoma reversal, however, fecal K^+^ concentration increased markedly to 49.6 and 60mmol/L after one and four weeks, respectively. These findings strongly suggest that the severe hyperkalemia in our patient was caused by the ileostomy and therefore significantly reduced colonic K^+^ secretion.

**Table 2:** Changes of fecal potassium concentration before and after stoma operation

**Fecal concentration**	**Before stoma reversal**			**After stoma reversal**	
Potassium, mmol/L	17	23.4	23.1	49.6	60

## Discussion

In this report, we present a case of severe hyperkalemia in a patient with ESRD and anuria undergoing chronic HD. Our patient had a well-controlled stable pre-dialysis K^+^ level prior to the colon diversion surgery. Since the severe hyperkalemia developed after the surgery and resolved after the restoration of bowel continuity, we assume that the ileostomy with colon diversion was the etiology of hyperkalemia. Augmented intestinal K^+^ excretion becomes a relevant quantitative phenomenon in ESRD, where the colon is able to partially substitute for the reduced renal K^+^ excretory capacity [8]. In our patient, colonic K^+^ excretion may have significantly contributed to the K^+^ homeostasis before the stoma operation. In the presence of ileostomy, however, there was no passage of stool in the large intestine and the colonic K^+^ excretion may have been reduced substantially, leading to severe hyperkalemia. The marked increase of fecal K^+^ concentration after the stoma reversal also strengthens our hypothesis.

To assess the colonic contribution to net gastrointestinal absorption and secretion of dietary K^+^ in a state without renal dysfunction, K^+^ output in ileostomy fluid was measured in several studies. Under ‘normal’ diet, K^+^ output was about 5mEq/day. Because K^+^ output in the stool of normal people averages about 9mEq/day, this would suggest that the normal colon secrets about 4mEq/day of K^+^[9]. In another study with slow marker perfusion, however, terminal ileal K^+^ was 9.3mEq/day and fecal K^+^ excretion was 4.7mEq/day, suggesting that the colon absorbed 4.6mEq of K^+^ per day [10]. Although these two studies do not agree, they show that the contribution of colon to K^+^ balance is relatively small. Net K^+^ secretion is found both in the proximal colon and distal colon in animals fed a high K^+^ diet [11]. The cellular mechanism of K^+^ secretion follows the general principle of ‘pump leak’ as applicable for many other types of ion secretion in epithelial tissues [12]. K^+^ is initially pumped into the cytosol by the basolateral Na^+^/K^+^-ATPase or via the basolateral secretory Na^+^/2Cl^-^/K^+^ co-transporter. In a second step, K^+^ leaves the cell into the gut lumen via apical K^+^ channels [13]. Dietary K^+^ loading was shown to stimulate an active K^+^ secretory process throughout the rat colon, which involved increased Na^+^/K^+^-ATPase-mediated K^+^ uptake across an amplified basolateral membrane, a rise in intra-cellular K^+^ concentration, and an increase in apical membrane K^+^ conductance [14].

Data obtained from patients with varying degrees of renal failure suggested that increased fecal K^+^ begins to contribute to maintenance of K^+^ balance when the creatinine clearance decreases below 5mL/minute; this is the same point at which residual nephrons develop their maximal capacity to secrete K^+^ into the urine [2]. In metabolic studies in patients with ESRD, the fecal K^+^ concentration was significantly higher than in healthy subjects [6,7]. However, the mechanism of enhanced fecal K^+^ excretion has not been completely understood. In *in vivo* studies using rectal dialysis technique, rectal K^+^ secretion was substantially greater in patients who are normokalemic with severely reduced renal function not yet established on dialysis and patients undergoing dialysis than in patients with normal renal function [8,15]. Enhanced rectal K^+^ secretion was independent of the transmucosal electrical potential difference, as well as the rate of Na^+^ absorption and the circulating level of plasma aldosterone, suggesting that the critical change in large intestinal K^+^ transport is an increase in active rather than passive K^+^ secretion [15]. In a further study using the same technique, rectal K^+^ secretion was almost threefold greater in patients with ESRD than in patents with normal renal function [5]. To study if the functional evidence for enhanced apical K^+^ permeability in patients with ESRD reflects increased expression of apical BK (so-called ‘big potassium’) channels, immunostaining using a specific antibody to the BK channel α-subunit was performed. This revealed greater levels of channel expression in patients with ESRD, suggesting that increased BK channel expression accounts for the increase in apical K^+^ permeability that occurs in the proximal rectum in ESRD. This adaptive change facilitates K^+^ movement into the lumen of the rectum and colon, and constitutes one of the factors underlying the ability of the large intestine to assume an accessory K^+^ excretory role in ESRD [5]. In a report by Simon *et al.* regarding a patient with ESRD, massive over-expression of colonic apical BK channels caused severe K^+^ secretory diarrhea. Even after performing ileostomy, plasma K^+^ concentrations remained low, suggesting that the K^+^ losses originated from the colon. In this patient, plasma K^+^ concentrations returned to normal following colectomy [16].

In terms of the locations of ischemic colitis, the ‘watershed’ areas, such as the splenic flexure and the transverse colon are known to be the most common locations. However, in patients with renal failure, especially those on hemodialysis, right-sided ischemic colitis is more commonly observed, as in our patient [17].

Some limitations of our report are the missing measurements of stool volume at the time of fecal K^+^ measurements. Additionally, we did not record our patient’s dietary potassium intake. However, the course of hyperkalemia before and after stoma reversal and the increasing fecal K^+^ concentration after stoma reversal strongly suggest the causal role of colon diversion surgery for the hyperkalemia.

## Conclusions

Our patient’s case demonstrates the importance of colonic K^+^ secretion for the maintenance of K^+^ homeostasis in patients with ESRD. In cases of hyperkalemia following colon diversion surgery in patients undergoing dialysis, a failing colonic K^+^ secretion should be considered as a potential etiology.

## Consent

Written informed consent was obtained from the patient for publication of this case report and any accompanying images. A copy of the written consent is available for review by the Editor-in-Chief of this journal.

## Competing interests

The authors declare that they have no competing interests.

## Authors’ contributions

NK, MD and MJK were all involved in the management of our patient and the analysis and interpretation of the data from our patient. NK and MJK wrote the manuscript. All authors read and approved the final manuscript.
